# Genetic evidence on the association between pulmonary function and cognitive impairment: A 2-sample Mendelian randomization study

**DOI:** 10.1097/MD.0000000000047457

**Published:** 2026-02-06

**Authors:** Xinran Cui, Weijie Zhai, Zixun Wang, Yanjiao Xu, Dongyao Fan, Qi Zhang, Li Sun

**Affiliations:** a Cognitive Center, Department of Neurology, The First Hospital of Jilin University, Jilin University, Changchun, China.

**Keywords:** cognitive function, dementia, Mendelian randomization, pulmonary function

## Abstract

Some observational studies have suggested that lower pulmonary function increases the risk of cognitive decline or dementia; however, the evidence remains inconclusive. We performed 2-sample Mendelian randomization (MR) analyses to investigate the potential associations between forced vital capacity (FVC) and a range of dementia- and cognition-related outcomes. FVC was selected as the primary indicator of pulmonary function because it is less effort- and cognition-dependent and better reflects overall lung capacity. Outcomes included 6 dementia types: all-cause dementia, Alzheimer disease (AD), dementia with lewy bodies, Parkinson disease dementia, frontotemporal dementia, and vascular dementia, and 6 cognitive domains, including intelligence, fluid intelligence (reasoning and problem-solving ability independent of acquired knowledge), cognitive performance, numeric memory, executive function, and prospective memory. All genetic associations were reported per 1-standard-deviation increase in genetically predicted FVC – expressed as log-odds ratios (log-ORs) for dementia outcomes and standard-deviation changes for cognitive outcomes. The inverse-variance weighted method was used as the primary analysis, complemented by MR-Egger, weighted median, weighted mode, simple mode and MR-PRESSO for sensitivity analyses. False discovery rate (FDR) correction, colocalization, and reverse MR analyses were also performed. This study provides genetic evidence supporting an association between reduced pulmonary function and cognitive impairment. Further studies are needed to clarify the underlying mechanisms.

Higher genetically predicted FVC was associated with a lower risk of AD (log-OR per 1-SD increase = −0.24; *P* = .002; FDR-adjusted *P* = .011). An inverse association was also observed with all-cause dementia (log-OR per 1-SD increase = −0.37; *P* = .031), but it did not remain significant after FDR correction (FDR-adjusted *P* = .094). No significant associations were observed for other dementia subtypes or cognitive outcomes. The results were robust in sensitivity analyses, with no significant findings in reverse MR. Colocalization analysis did not support shared causal variants between FVC and AD (PP.H_4_.abf <0.75).

## 1. Introduction

With the aging of the global population, the incidence of dementia is forecasted to rise substantially in the years ahead.^[1]^ Dementia is primarily characterized by progressive cognitive decline, especially memory impairment, which significantly reduces patients’ quality of life and creates a substantial burden on families and society.^[2]^ Currently, no effective intervention exists to reverse or halt disease progression. Thus, identifying modifiable risk factors and preventive strategies is critical to reducing the incidence of dementia. Several cohort studies have reported a relationship between reduced pulmonary function and cognitive performance.^[3–5]^ For instance, the Rotterdam Study (n = 3941) found that individuals with preserved ratio impaired spirometry (PRISm), a specific pattern of pulmonary impairment, demonstrated poorer global cognitive function. Similarly, the Atherosclerosis Risk in Communities study observed that impaired pulmonary function was linked to lower initial cognitive performance and a greater likelihood of dementia over time.^[3,6]^ Nonetheless, other studies have not found significant connections and the overall evidence remains inconsistent.^[7]^ Considering that observational studies may suffer from remaining confounding factors and the possibility of reverse causation, we utilized Mendelian randomization (MR) to investigate the potential association between pulmonary function and dementia or cognitive performance at the genetic level. Because genetic variants are randomly allocated at conception, they are largely unaffected by disease status and can reduce confounding, thereby improving the reliability of causal inference.^[8]^

In this study, forced vital capacity (FVC) was selected as the exposure variable. Other commonly used spirometric measures, such as forced expiratory volume in 1 second (FEV_1_), the FEV_1_/FVC ratio, and peak expiratory flow (PEF), were not included because they depend more heavily on participants’ comprehension and task execution, which may introduce bias into causal inference. In contrast, FVC provides a more stable and less cognitively dependent measure of overall lung capacity.

In MR, genetic variants significantly associated with the exposure are used as instrumental variables (IVs) to infer potential relationships between traits.

**Figure 1.**
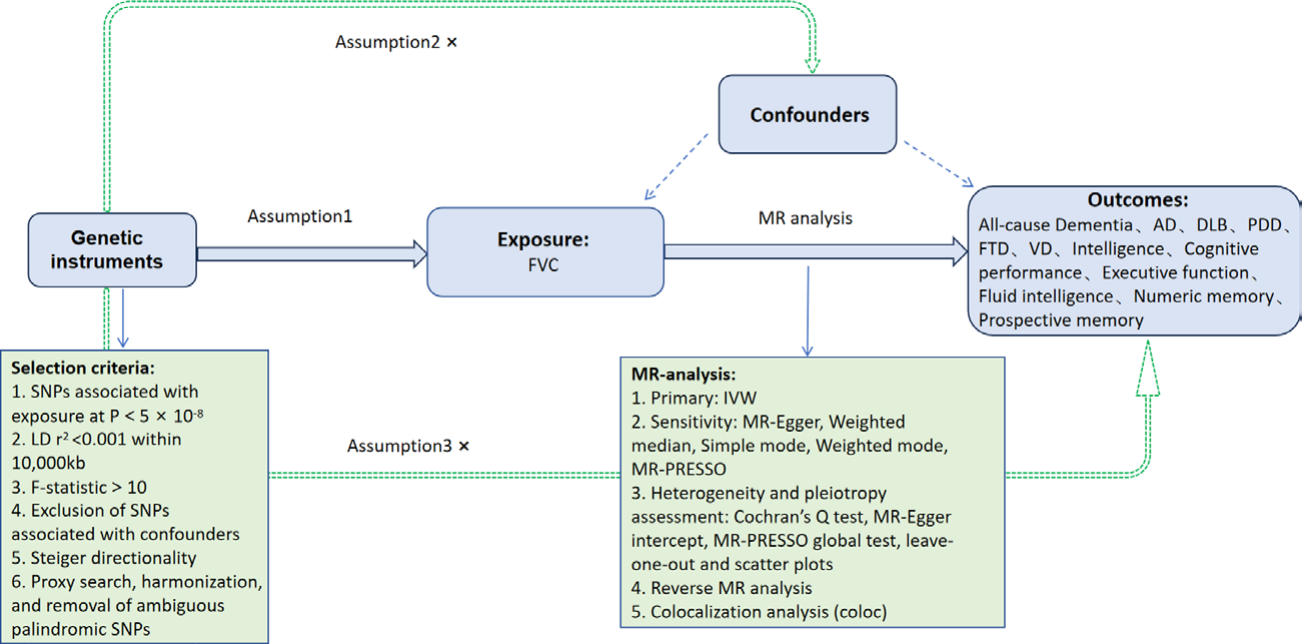
Diagram illustrating the 2-sample MR framework used in this study. Genetic variants significantly associated with FVC were selected as IVs under 3 key assumptions: (1) relevance, (2) independence from confounders, and (3) exclusion restriction. The MR framework incorporated sensitivity analyses, heterogeneity and pleiotropy assessments, reverse MR, and colocalization analysis. MR = Mendelian randomization, FVC = forced vital capacity, IVs = instrumental variables, SNPs = single-nucleotide polymorphisms.

The selection of IVs relied on 3 fundamental assumptions (Fig. 1): relevance – the IVs are strongly associated with the exposure (pulmonary function); independence from confounders – the IVs are independent of any potential confounders of the exposure–outcome association; and exclusion restriction – the IVs influence the outcome (dementia or cognitive function) only through the exposure, with no alternative biological pathways.^[9]^

Only IVs meeting these assumptions were included in the main analysis to minimize bias and strengthen the validity of inference.^[9]^ In this study, IVs were represented by single-nucleotide polymorphisms (SNPs).

## 2. Resources and approach

### 2.1. Data sources

All summary-level genome-wide association study (GWAS) data were obtained from publicly available repositories, including UK Biobank, FinnGen, the EBI GWAS Catalog and the IEU OpenGWAS Project.

All datasets were derived from participants of European ancestry to minimize bias due to population stratification. For cognitive outcomes, the GWAS summary statistics for intelligence were derived from meta-analyses integrating genome-wide association data on general cognitive ability across multiple neurocognitive assessments. Fluid intelligence, reflecting reasoning and problem-solving ability independent of prior knowledge, was assessed by the number of correct responses to 13 logic and reasoning questions within a limited time frame. Cognitive performance represents a composite indicator of general cognitive ability derived from multiple cognitive tests. Numeric memory was evaluated by recording the maximum number length participants could accurately recall. Prospective memory was assessed by examining whether participants could remember and execute previously instructed tasks while completing other activities. Executive function was assessed using the Trail Making Test and the backward Verbal Digit Span task. As all datasets were publicly available and ethically approved in the original studies, no additional ethical approval was required for this secondary analysis. Sample sizes, GWAS identifiers, and summary-level data sources for each trait are detailed in Table 1.

**Table 1:** Summary information of GWAS datasets used in the MR analyses.

Traits	ID	Data source	Year	Sample size	Cases, n	Controls, n	Population
Pulmonary function (exposures)							
FVC	ieu-b-105	UK Biobank	2021	353,315	NA	NA	European
Dementia (outcomes)							
All-cause dementia	finn-b-F5_DEMENTIA	FinnGen	2021	216,771	7284	209,487	European
AD	ebi-a-GCST90027158	EBI GWAS Catalog	2022	487,511	39,106	46,828	European
DLB	ebi-a-GCST90001390	EBI GWAS Catalog	2021	6618	2591	4027	European
PDD	finn-b-PD_DEMENTIA	FinnGen	2021	216,895	267	216,628	European
FTD	Ferrari et al., Lancet Neurol 2014 (PMID: 24943344)	NA	2014	12,928	3526	9402	European
VD	finn-b-F5_VASCDEM	FinnGen	2021	212,389	881	211,508	European
Cognitive function (outcomes)							
Intelligence	ebi-a-GCST006250	IEU OpenGWAS	2018	269,867	NA	NA	European
Fluid intelligence	ukb-b-5238	UK Biobank	2018	149,051	NA	NA	European
Cognitive performance	ebi-a-GCST006572	EBI GWAS Catalog	2018	257,841	NA	NA	European
Numeric memory	ukb-b-16872	UK Biobank	2018	48,080	NA	NA	European
Prospective memory	ukb-b-4282	UK Biobank	2018	152,605	NA	NA	European
Executive function	ebi-a-GCST90020168	EBI GWAS Catalog	2021	1297	NA	NA	European

FVC was treated as a continuous exposure variable. Dementia outcomes were binary traits, and cognitive outcomes were continuous traits. All traits represent genetically predicted liabilities derived from GWAS summary statistics.

AD = Alzheimer disease, DLB = dementia with Lewy bodies, FVC = forced vital capacity, FTD = frontotemporal dementia, GWAS = genome-wide association study, NA = not applicable, PDD = Parkinson disease dementia, VD = vascular dementia.

### 2.2. Study of Mendelian randomization

This study utilized an MR method involving 2 samples to examine the potential associations between genetically predicted pulmonary function and the risks of dementia and cognitive impairment. Analyses were based on publicly available GWAS summary statistics.

The inverse-variance weighted (IVW) method served as the primary analysis, which combines the ratio estimates from all IVs to obtain an overall pooled effect. This method provides the most statistically efficient estimates when all IVs are valid and no directional horizontal pleiotropy is present.

To test the robustness of the results, complementary MR methods – including MR-Egger regression, weighted median, weighted mode, and simple mode – were performed.

Cochran Q statistic was used to assess heterogeneity among SNP-specific estimates. When significant heterogeneity was detected (Q *P* <.05), random-effects IVW models were adopted to obtain more reliable estimates.^[10]^

Horizontal pleiotropy was further evaluated using the MR-Egger regression and the MR-PRESSO global test.^[11]^

The MR-Egger intercept was used to assess directional pleiotropy; a *P*-value >.05 indicated no strong evidence of directional pleiotropy^[11,12]^

The MR-PRESSO global test was applied in all analyses to detect potentially influential outlier SNPs (1000 simulations were used to derive the *P*-value). Identified outliers were removed before reestimation.^[11]^

To address multiple testing, false discovery rate (FDR) correction was applied across outcome families (dementia and cognitive outcomes, respectively) to minimize false-positive findings. Results with FDR <0.05 were considered statistically significant.

Leave-one-out analyses were conducted to assess whether any single SNP disproportionately influenced the MR estimates. Scatter plots were generated to visualize SNP-specific associations and to assess the overall direction and consistency of the MR estimates.

In addition, reverse MR analyses were performed to examine the potential direction of association from dementia- and cognition-related traits to pulmonary function (FVC).

All MR estimates were reported on a unified scale. The FVC GWAS was originally standardized to standard-deviation (SD) = 0.984 and rescaled to SD = 1 for comparability. For binary outcomes (dementia), results were expressed as log-ORs per 1-SD increase in genetically predicted FVC. For continuous cognitive traits (already z-standardized in the original GWAS), effects were expressed as SD changes per 1-SD increase in the exposure.

### 2.3. Selection of instrumental variables

We chose SNPs significantly linked to the exposure by using a criterion of *P *<5 × 10^−8^. To avoid linkage disequilibrium (LD) and maintain the independence of IVs, LD clumping was conducted using an *R^2^* threshold <0.001 within a 10,000 kb window.^[13]^

To ensure sufficient instrument strength, only SNPs with *F*-statistics >10 were retained.^[14]^ Applying the formula F = [K × (1 − *R^2^*)]/ [(N − K − 1) × *R^2^*], F-statistic was computed, where *R^2^* denotes the extent to which the genetic instruments explain the variance in the exposure, N refers to the exposure GWAS sample size, and K represents the quantity of SNPs utilized.^[14]^ The overall instrument strength was summarized using the total variance explained (*R^2^*_total) and the median F-statistic (F_median), which are reported in Tables S1 and 2, Supplemental Digital Content, https://links.lww.com/MD/R288.

In addition, we queried the LDlink platform and the NHGRI-EBI GWAS Catalog to identify phenotypic associations of the remaining SNPs and excluded variants showing genome-wide significant associations with potential confounders (*P* <5 × 10^−^8).

Based on extensive literature, SNPs associated with the following categories were removed: education and cognition (educational attainment; cognitive ability traits such as intelligence and cognitive performance; dementia-related traits such as Alzheimer disease (AD) and Parkinson disease dementia); socioeconomic status (indicators of occupational and social position); lifestyle factors (smoking and physical activity); mental health (depression, subjective well-being, life satisfaction, positive affect, autism spectrum disorder, schizophrenia, and other psychiatric disorders); cardiovascular, cerebrovascular, and metabolic traits/risk factors (coronary heart disease, myocardial infarction, stroke, blood pressure, diabetes, and blood lipids); anthropometric/adiposity traits (body mass index and central adiposity measures such as waist circumference, hip circumference, and waist-to-hip ratio); sleep traits (sleep duration and insomnia).

After this phenome-wide screening, 190 SNPs were excluded, leaving 107 independent SNPs for subsequent analyses (Table S3, Supplemental Digital Content, https://links.lww.com/MD/R288).

During outcome data extraction, proxy SNP searching was enabled (proxies = TRUE, *r^2^* >0.8, MAF threshold = 0.01) to minimize variant loss. Subsequently, allele harmonization was performed to align effect alleles between exposure and outcome datasets, and ambiguous palindromic SNPs were removed.

In addition, Steiger directionality tests were performed to verify that the causal direction flowed from the exposure to the outcome. The proportion of SNPs passing this test (*Steiger_pass*) was reported in the MR results table to ensure the validity of instrument selection.

The remaining SNPs were retained for the main MR analyses.

### 2.4. Colocalization analysis

Colocalization analysis was performed using the coloc R package to assess whether pulmonary function (FVC) and dementia-related traits share a common causal variant within ± 50 kb around the top associated SNPs. Under a Bayesian framework, 5 mutually exclusive hypotheses (H0–H4) were tested, with the posterior probability for H4 (PP.H4) representing the likelihood of a shared causal variant between the traits. Evidence for colocalization was considered when PP.H4 exceeded 0.75, consistent with commonly used thresholds in previous studies. All analyses were conducted in R 4.3.3.

### 2.5. Ethics declaration

This study used only summary-level genetic data obtained from publicly accessible databases. All original GWAS contributing to the analyses had received ethical approval from their respective institutional review boards, and informed consent had been obtained from all participants. The present research followed the STROBE-MR reporting guidelines for MR studies.^[15]^ Because only secondary analyses of publicly available, de-identified data were conducted, no additional ethical approval or informed consent was required.

## 3. Result

### 3.1. The association between pulmonary function and dementia and cognitive function

Two-sample MR analyses were performed to examine the associations between genetically predicted FVC and multiple dementia and cognitive outcomes. The IVW method served as the primary estimator. When significant heterogeneity was detected (Cochran *Q P* <.05), random-effects IVW models were applied.

In analyses with dementia as the outcome, higher genetically predicted FVC showed a significant association with lower risk of AD (log-OR per 1-SD increase = −0.24; *P* = .002; FDR-adjusted *P* = .011). An inverse association was also observed with all-cause dementia (log-OR per 1-SD increase = −0.37; *P* = .031), although this did not remain significant after FDR correction (FDR-adjusted *P* = .094) (Table 2). No significant associations were detected for other dementia subtypes or any of the 6 cognitive traits.

**Table 2:** Two sample MR analyses for the associations between genetically predicted FVC and dementia outcomes.

Exposure	Outcome	Method	nSNPs	log-OR (per 1-SD increase)	SE	*P*	FDR-adjusted P	95% CI (log-OR)
FVC	AD	IVW	91	−0.238	0.077	.002	0.011	−0.388 to −0.088
FVC	All-cause dementia	IVW	93	−0.371	0.173	.031	0.094	−0.709 to −0.033

All MR estimates are expressed as log-ORs per 1-SD increase in genetically predicted FVC, with corresponding standard errors and 95% confidence intervals. Random-effects IVW models were applied when heterogeneity was detected. FDR-adjusted *P*-values account for multiple testing.

CI = confidence interval, FDR = false discovery rate, FVC = forced vital capacity, IVW = inverse-variance weighted, MR = Mendelian randomization, OR = odds ratio, SD = standard deviation, SE = standard error.

### 3.2. Sensitivity and reverse MR analyses

For the FVC–AD association, moderate heterogeneity was detected (IVW/MR-Egger *P* <.05); therefore, random-effects IVW estimates were reported. The MR-Egger intercept was close to zero (intercept = 0.001, *P* = .814), suggesting no evidence of directional horizontal pleiotropy (Table 3).

**Table 3:** Heterogeneity and horizontal pleiotropy tests for MR analyses.

Exposure	Outcome	Heterogeneity test				Pleiotropic test		
		Method	*Q* statistic	*Q* df	*P*	Method	Egger intercept	*P*
FVC	AD	MR-Egger	116.445	89	.027	MR-Egger	0.001	.814
		IVW	116.519	90	.032			
FVC	All-cause dementia	MR-Egger	106.974	91	.121	MR-Egger	0.008	.316
		IVW	108.167	92	.120			

Cochran *Q* test evaluates heterogeneity across SNP-specific estimates; *P* < .05 indicates significant heterogeneity, for which random-effects IVW models were applied. The MR-Egger intercept test assesses directional horizontal pleiotropy; nonsignificant *P*-values (*P* >.05) indicate no evidence of directional pleiotropy.

AD = Alzheimer disease, FVC = forced vital capacity, IVW = inverse-variance weighted, MR = Mendelian randomization, Q df = degrees of freedom for *Q* statistic.

**Figure 2.**
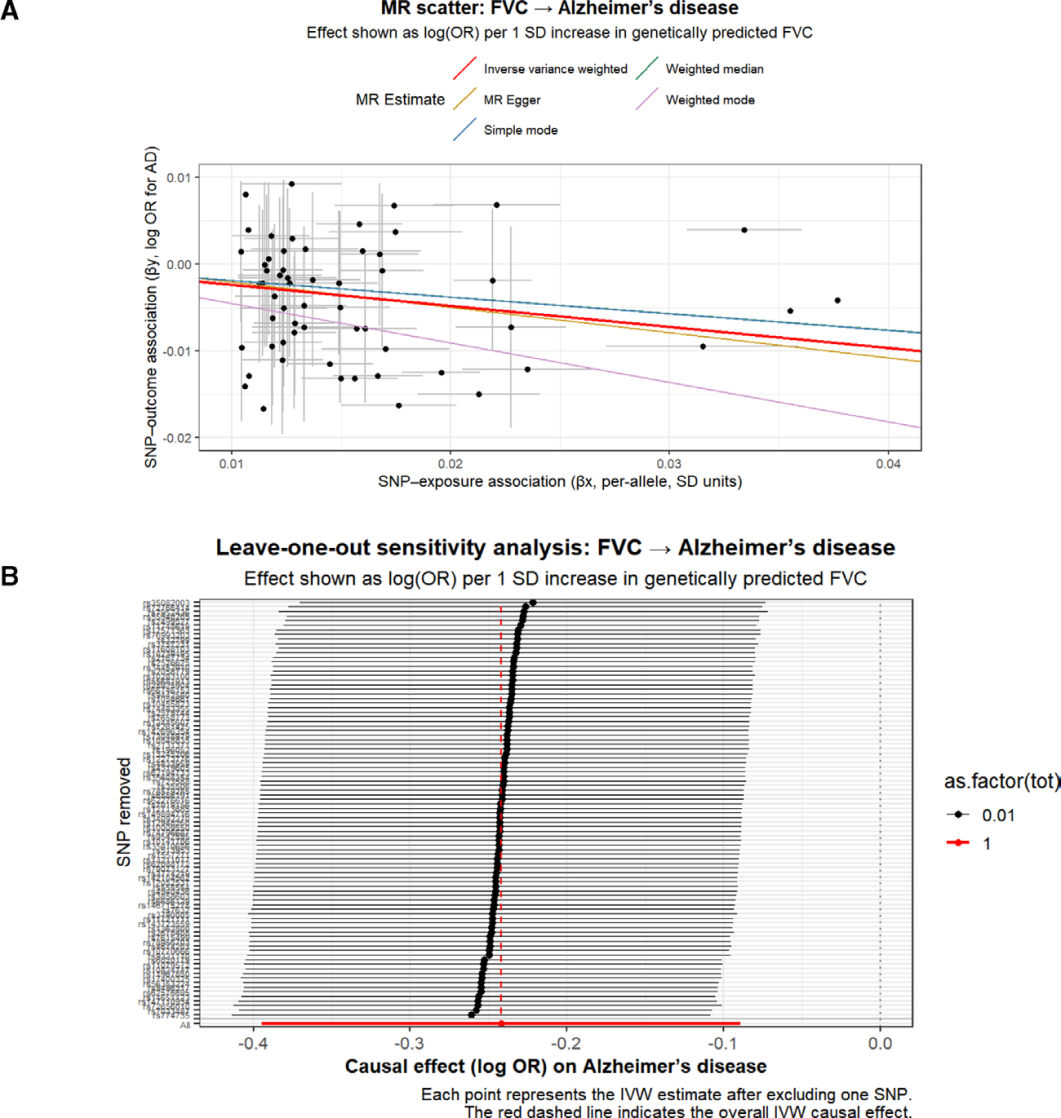
MR results for the association between FVC and AD. (A) Scatter plot showing SNP–exposure and SNP–outcome associations, with regression lines for different MR estimators. (B) Leave-one-out sensitivity analysis illustrating the causal effect (log-odds ratio [log-OR]) per 1-SD increase in genetically predicted FVC. AD = Alzheimer disease, FVC = forced vital capacity, MR = Mendelian randomization, OR = odds ratio, SD = standard deviation, SNP = single-nucleotide polymorphism.

The MR-PRESSO global test detected 1 potential outlier for the FVC–AD association (Global test *P* = .014), which was removed before the main analysis. After correction, no significant distortion was detected (distortion test *P* = .829), indicating that outlier removal did not materially influence the overall estimates. In contrast, no outliers were identified for the FVC–overall dementia association. Sensitivity analyses using the weighted median, weighted mode, and MR-Egger methods yielded consistent directions, supporting the robustness of the primary results. Steiger directionality tests indicated that 98.9% of SNPs supported the direction from FVC to AD and 100% for overall dementia. All IVs were sufficiently strong (*R*^2^_total = 0.0073; *F*_median = 22.7 for the FVC–AD analysis and *R*^2^_total = 0.0077; *F*_median = 22.8 for the FVC–overall dementia analysis), indicating a low risk of weak-instrument bias. Full details are provided in Table S1, Supplemental Digital Content, https://links.lww.com/MD/R288. Reverse MR analyses yielded null findings (Tables S2, 4, and 5, Supplemental Digital Content, https://links.lww.com/MD/R288), and scatter and leave-one-out plots are presented in Figure 2.

### 3.3. Genetic variant loci

**Figure 3.**
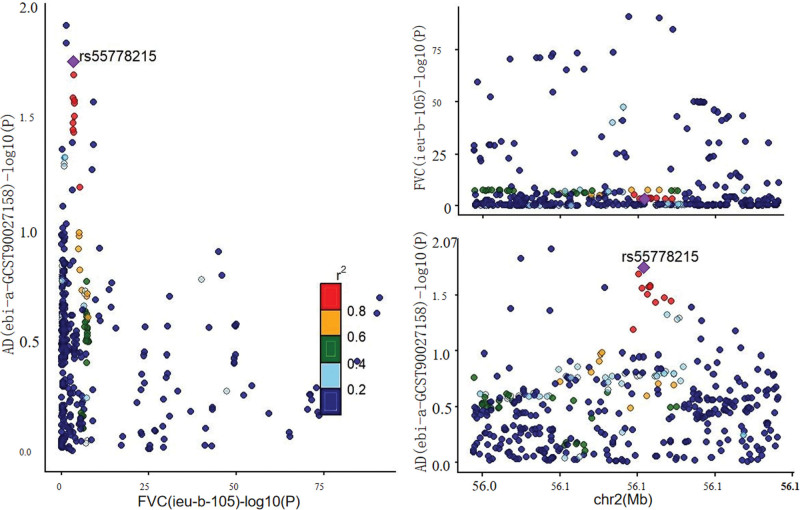
Colocalization analysis between FVC and AD. Regional plots of association signals for FVC (ieu-b-105) and AD (ebi-a-GCST90027158). AD = Alzheimer disease, FVC = forced vital capacity.

Colocalization analysis was performed between FVC and AD, the only association that reached FDR significance, to explore potential shared genetic architecture. The results provided no evidence of a shared causal variant between FVC and AD (PP.H_4_ = 0.010 <0.75), suggesting that the observed MR association is unlikely to be driven by the same underlying genetic signal (Fig. 3).

## 4. Discussion

Existing research on the relationship between pulmonary function and dementia or cognitive performance has largely relied on observational evidence. Clinical studies have reported that obstructive sleep apnea, chronic obstructive pulmonary disease (COPD), asthma, and nocturnal hypoxemia are associated with an increased risk of cognitive decline and dementia.^[16,17]^ Prospective cohort studies further indicate that a diagnosis of COPD is linked to a higher risk of mild cognitive impairment (MCI), with longer disease duration corresponding to greater risk, suggesting a dose–response relationship.^[18–20]^ Given that respiratory disturbances are closely related to lung capacity, some studies have proposed that reduced pulmonary function may contribute to dementia risk and lower cognitive performance. Longitudinal evidence also suggests that declining lung function is associated not only with an elevated risk of MCI but may also accelerate progression from MCI to dementia.^[21]^ For example, a long-term follow-up study reported that lower midlife pulmonary function, indexed by FEV_1_/height,^[2]^ predicted poorer memory, processing speed, and executive function 23 years later, as well as a higher risk of developing MCI and dementia.^[22]^ Consistently, a recent large-scale prospective study from the UK Biobank, including more than 330,000 participants with a mean follow-up of 12.8 years, found that lower levels of FEV_1_, FVC, and PEF were significantly associated with an increased risk of AD.^[23]^

From a genetic perspective, MR studies investigating the relationship between pulmonary function and dementia risk remain limited. Early MR analyses did not find evidence supporting a causal association between pulmonary function traits (such as FVC, FEV_1_, and PEF) and AD, suggesting that previously observed associations in observational cohorts may partly reflect unmeasured confounding factors.^[24–26]^ More recently, however, a 2-sample MR study reported a robust inverse association between genetically predicted FEV_1_ and AD risk, providing new genetic evidence for a potential link between lung function and dementia. Consistent with this emerging literature, our MR analysis also observed that higher genetically predicted FVC was associated with a lower risk of AD. In addition, we further incorporated Steiger directionality tests and reverse MR analyses to evaluate the robustness of the causal direction. Both approaches consistently supported a direction of effect from FVC to AD rather than the reverse. We also performed colocalization analysis to assess whether FVC and AD might be driven by the same underlying genetic variants. The results did not identify shared causal loci, suggesting that their genetic associations are more likely attributable to distinct biological signals.

The biological pathways linking reduced pulmonary function to dementia remain incompletely understood. Current evidence suggests that impaired lung function may influence the brain through systemic inflammation, immune dysregulation, and neuroendocrine alterations – mechanisms often referred to as the “lung–brain axis.”^[27,28]^ Neuropathological studies report that COPD is associated with cerebrovascular abnormalities,^[29]^ and that lower lung capacity and respiratory muscle strength relate to neuronal loss, AD pathology, and macroscopic infarcts.^[30]^ Recent findings further indicate that reduced pulmonary function correlates with higher burdens of AD pathology and vascular lesions, including cerebral atherosclerosis and cerebral amyloid angiopathy.^[21]^ Neuroimaging studies also show that poorer lung function is linked to smaller brain volumes and greater white matter hyperintensity burden.^[31–34]^

## 5. Strengths and Limitations

This study has several notable strengths. First, to reduce potential bias arising from the effort dependence of spirometric measures, we selected FVC – which is less influenced by cognitive ability and task performance – as the genetic exposure, thereby minimizing behavioral coupling between pulmonary function and cognition. Second, we implemented a more stringent and comprehensive instrument-selection process than previous studies. In addition to applying strict genome-wide significance and LD thresholds, we conducted an extensive phenome-wide screening to exclude SNPs associated with education, cognition, socioeconomic status, lifestyle factors, mental health conditions, cardiometabolic diseases, adiposity traits, and sleep characteristics, thereby minimizing potential pleiotropy and residual confounding. Third, we expanded the outcome spectrum substantially by assessing 6 dementia subtypes and 6 cognitive phenotypes, enabling a more systematic evaluation of the genetic relationship between pulmonary function and multiple domains of cognitive performance. Finally, we incorporated multiple layers of validation – including Steiger directionality testing, reverse MR, and colocalization analysis – to reinforce the robustness of causal inference and to evaluate the possibility of shared genetic signals between FVC and AD.

Several limitations should be acknowledged. First, the MR analyses were conducted exclusively in individuals of European ancestry, and the generalizability of the findings to other populations remains uncertain. Second, although we attempted to minimize effort dependence, FVC measurement still involves participant cooperation, and genetic variants related to FVC may partly overlap with cognitive traits, potentially introducing bias. Third, despite extensive phenome-wide screening, residual pleiotropy cannot be fully excluded. Finally, although FDR correction was applied, the possibility of false-positive results cannot be entirely ruled out.

## 6. Conclusion

This study provides genetic evidence suggesting that lower pulmonary function, as reflected by FVC, may be associated with a higher risk of AD. The results highlight the potential relevance of maintaining pulmonary health for cognitive aging, although causal mechanisms remain uncertain and require further investigation in future studies.

## Author contributions

**Conceptualization:** Xinran Cui, Li Sun.

**Data curation:** Xinran Cui, Weijie Zhai.

**Formal analysis:** Weijie Zhai.

**Funding acquisition:** Li Sun.

**Investigation:** Zixun Wang, Yanjiao Xu, Dongyao Fan, Qi Zhang.

**Methodology:** Weijie Zhai, Zixun Wang.

**Project administration:** Xinran Cui.

**Software:** Weijie Zhai, Zixun Wang.

**Supervision:** Li Sun.

**Validation:** Xinran Cui, Weijie Zhai, Zixun Wang, Yanjiao Xu, Dongyao Fan, Qi Zhang, Li Sun.

**Visualization:** Xinran Cui.

**Writing – original draft:** Xinran Cui, Weijie Zhai.

**Writing – review & editing:** Xinran Cui, Zixun Wang, Yanjiao Xu, Dongyao Fan, Qi Zhang, Li Sun.

## Supplementary Material



## References

[1] GBD 2019 Dementia Forecasting Collaborators. Estimation of the global prevalence of dementia in 2019 and forecasted prevalence in 2050: an analysis for the global burden of disease study 2019. Lancet Public Health. 2022;7:e105–25. 34998485 10.1016/S2468-2667(21)00249-8 PMC8810394

[2] Scheltens P De Strooper B Kivipelto M. Alzheimer’s disease. Lancet. 2021;397:1577–90. 33667416 10.1016/S0140-6736(20)32205-4 PMC8354300

[3] Shrestha S Zhu X London SJ. Association of lung function with cognitive decline and incident dementia in the atherosclerosis risk in communities study. Am J Epidemiol. 2023;192:1637–46. 37392093 10.1093/aje/kwad140 PMC11292409

[4] Richards M Strachan D Hardy R Kuh D Wadsworth M. Lung function and cognitive ability in a longitudinal birth cohort study. Psychosom Med. 2005;67:602–8. 16046374 10.1097/01.psy.0000170337.51848.68

[5] Gilsanz P Mayeda ER Flatt J Glymour MM Quesenberry CP Jr Whitmer RA. Early midlife pulmonary function and dementia risk. Alzheimer Dis Assoc Disord. 2018;32:270–5. 29543604 10.1097/WAD.0000000000000253 PMC6138567

[6] Pathan SS Gottesman RF Mosley TH Knopman DS Sharrett AR Alonso A. Association of lung function with cognitive decline and dementia: the atherosclerosis risk in communities (ARIC) study. Eur J Neurol. 2011;18:888–98. 21244584 10.1111/j.1468-1331.2010.03340.x PMC3092022

[7] Sibbett RA Russ TC Allerhand M Deary IJ Starr JM. Physical fitness and dementia risk in the very old: a study of the Lothian Birth Cohort 1921. BMC Psychiatry. 2018;18:285. 30180830 10.1186/s12888-018-1851-3 PMC6123983

[8] Davies NM Holmes MV Davey Smith G. Reading Mendelian randomisation studies: a guide, glossary, and checklist for clinicians. BMJ. 2018;362:k601. 30002074 10.1136/bmj.k601 PMC6041728

[9] Wang Y Ge Y Yan W Wang L Zhuang Z He D. The association between walking speeds and osteoarthritis risk based on the National Health and Nutrition Examination Survey and Mendelian randomization study. Public Health. 2025;247:105867. 40829545 10.1016/j.puhe.2025.105867

[10] Kuang S Ma X Sun L. Exploring the association between immune cell phenotypes and osteoporosis mediated by inflammatory cytokines: insights from GWAS and single-cell transcriptomics. Immunotargets Ther. 2025;14:227–46. 40125424 10.2147/ITT.S510102 PMC11927574

[11] Qu J Zhang L. The gut microbiome and ovarian cysts: a mendelian randomization study. J Ovarian Res. 2025;18:188. 40826477 10.1186/s13048-025-01767-3 PMC12359854

[12] Xiao P Feng X Fan Y Wang Y Ye R Qiu F. The causal relationship between rheumatoid arthritis and the risk of leg ulcers: a mendelian randomization study. Geriatr Nurs. 2025;66(Pt A):103583. 40829530 10.1016/j.gerinurse.2025.103583

[13] Ji T Lv Y Yang J Diao X Gu J. Association between depression and asthma: insight from observational and genetic evidence. BMC Psychiatry. 2025;25:786. 40796827 10.1186/s12888-025-07245-w PMC12341131

[14] Cheng G Xu L Li H. Causal links between personality disorders and schizophrenia: a Mendelian randomization study. Medicine (Baltim). 2025;104:e42532. 10.1097/MD.0000000000042532 PMC12091683 40388757

[15] Skrivankova VW Richmond RC Woolf BAR. Strengthening the reporting of observational studies in epidemiology using mendelian randomisation (STROBE-MR): explanation and elaboration. BMJ. 2021;375:n2233. 34702754 10.1136/bmj.n2233 PMC8546498

[16] Legault J Thompson C Moullec G. Age- and sex-specific associations between obstructive sleep apnea risk and cognitive decline in middle-aged and older adults: a 3-year longitudinal analysis of the Canadian longitudinal study on aging. Sleep Med. 2023;112:77–87. 37832163 10.1016/j.sleep.2023.09.029

[17] Ji X Zhu Y Ahmadizar F. Cognitive decline before and after incident chronic respiratory disease. Geroscience. 2025;47:6699–710. 40540151 10.1007/s11357-025-01754-y PMC12638625

[18] Singh B Mielke MM Parsaik AK. A prospective study of chronic obstructive pulmonary disease and the risk for mild cognitive impairment. JAMA Neurol. 2014;71:581–8. 24637951 10.1001/jamaneurol.2014.94 PMC4020948

[19] Kakkera K Padala KP Kodali M Padala PR. Association of chronic obstructive pulmonary disease with mild cognitive impairment and dementia. Curr Opin Pulm Med. 2018;24:173–8. 29232279 10.1097/MCP.0000000000000458

[20] Rusanen M Ngandu T Laatikainen T Tuomilehto J Soininen H Kivipelto M. Chronic obstructive pulmonary disease and asthma and the risk of mild cognitive impairment and dementia: a population based CAIDE study. Curr Alzheimer Res. 2013;10:549–55. 23566344 10.2174/1567205011310050011

[21] Wang J Dove A Song R. Poor pulmonary function is associated with mild cognitive impairment, its progression to dementia, and brain pathologies: a community-based cohort study. Alzheimers Dement. 2022;18:2551–9. 35184372 10.1002/alz.12625 PMC10078691

[22] Vidal JS Aspelund T Jonsdottir MK. Pulmonary function impairment may be an early risk factor for late-life cognitive impairment. J Am Geriatr Soc. 2013;61:79–83. 23311554 10.1111/jgs.12069 PMC3545414

[23] Zheng YN Qiu P Luo HH Chen RJ Wang XQ Chen PJ. Association of pulmonary function with the risk of incident Alzheimer’s disease: a prospective cohort and Mendelian randomization study. Aging Clin Exp Res. 2025;37:244. 40802026 10.1007/s40520-025-03151-z PMC12350532

[24] Li J Zhao L Ding X Cui X Qi L Chen Y. Obstructive sleep apnea and the risk of Alzheimer’s disease and Parkinson disease: a Mendelian randomization study OSA, Alzheimer’s disease and Parkinson disease. Sleep Med. 2022;97:55–63. 35724440 10.1016/j.sleep.2022.06.004

[25] Higbee D Granell R Walton E Korologou-Linden R Davey Smith G Dodd J. Examining the possible causal relationship between lung function, COPD and Alzheimer’s disease: a Mendelian randomisation study. BMJ Open Respir Res. 2021;8:e000759. 10.1136/bmjresp-2020-000759 PMC8264898 34233891

[26] Russ TC Harris SE Batty GD. Pulmonary function and risk of alzheimer dementia: two-sample mendelian randomization study. Chest. 2021;160:274–6. 33321123 10.1016/j.chest.2020.11.056

[27] Ma YH Shen LX Li YZ. Lung function and risk of incident dementia: a prospective cohort study of 431,834 individuals. Brain Behav Immun. 2023;109:321–30. 36796705 10.1016/j.bbi.2023.02.009

[28] Russ TC Starr JM Stamatakis E Kivimäki M Batty GD. Pulmonary function as a risk factor for dementia death: an individual participant meta-analysis of six UK general population cohort studies. J Epidemiol Community Health. 2015;69:550–6. 25691274 10.1136/jech-2014-204959

[29] Cleutjens FA Spruit MA Beckervordersandforth J. Presence of brain pathology in deceased subjects with and without chronic obstructive pulmonary disease. Chron Respir Dis. 2015;12:284–90. 26033836 10.1177/1479972315588005

[30] Buchman AS Yu L Wilson RS. Post-mortem brain pathology is related to declining respiratory function in community-dwelling older adults. Front Aging Neurosci. 2015;7:197. 26539108 10.3389/fnagi.2015.00197 PMC4612667

[31] Shrestha S Zhu X Sullivan KJ. Lung function and brain MRI outcomes in the atherosclerosis risk in communities neurocognitive study. J Alzheimers Dis. 2024;100:297–308. 38848187 10.3233/JAD-240162 PMC11223445

[32] Frenzel S Bis JC Gudmundsson EF. Associations of pulmonary function with MRI brain volumes: a coordinated multi-study analysis. J Alzheimers Dis. 2022;90:1073–83. 36213999 10.3233/JAD-220667 PMC9712227

[33] Sachdev PS Anstey KJ Parslow RA. Pulmonary function, cognitive impairment and brain atrophy in a middle-aged community sample. Dement Geriatr Cogn Disord. 2006;21:300–8. 16484809 10.1159/000091438

[34] Yin M Wang H Hu X Li X Fei G Yu Y. Patterns of brain structural alteration in COPD with different levels of pulmonary function impairment and its association with cognitive deficits. BMC Pulm Med. 2019;19:203. 31699064 10.1186/s12890-019-0955-y PMC6839173

